# Repair Injured Heart by Regulating Cardiac Regenerative Signals

**DOI:** 10.1155/2016/6193419

**Published:** 2016-10-09

**Authors:** Wen-Feng Cai, Guan-Sheng Liu, Lei Wang, Christian Paul, Zhi-Li Wen, Yigang Wang

**Affiliations:** ^1^Department of Pathology & Lab Medicine, College of Medicine, University of Cincinnati, Cincinnati, OH 45267, USA; ^2^Department of Pharmacology & Cell Biophysics, College of Medicine, University of Cincinnati, Cincinnati, OH 45267, USA; ^3^Infectious Disease Hospital, Nanchang University, Nanchang, Jiangxi 330002, China

## Abstract

Cardiac regeneration is a homeostatic cardiogenic process by which the sections of malfunctioning adult cardiovascular tissues are repaired and renewed employing a combination of both cardiomyogenesis and angiogenesis. Unfortunately, while high-quality regeneration can be performed in amphibians and zebrafish hearts, mammalian hearts do not respond in kind. Indeed, a long-term loss of proliferative capacity in mammalian adult cardiomyocytes in combination with dysregulated induction of tissue fibrosis impairs mammalian endogenous heart regenerative capacity, leading to deleterious cardiac remodeling at the end stage of heart failure. Interestingly, several studies have demonstrated that cardiomyocyte proliferation capacity is retained in mammals very soon after birth, and cardiac regeneration potential is correspondingly preserved in some preadolescent vertebrates after myocardial infarction. There is therefore great interest in uncovering the molecular mechanisms that may allow heart regeneration during adult stages. This review will summarize recent findings on cardiac regenerative regulatory mechanisms, especially with respect to extracellular signals and intracellular pathways that may provide novel therapeutics for heart diseases. Particularly, both* in vitro* and* in vivo* experimental evidences will be presented to highlight the functional role of these signaling cascades in regulating cardiomyocyte proliferation, cardiomyocyte growth, and maturation, with special emphasis on their responses to heart tissue injury.

## 1. Introduction

The mammalian heart is generally considered a circulatory pump with poor reparative and regenerative capabilities due to the cell-cycle withdrawal and mitosis cessation of cardiomyocytes soon after birth. A recent study identified a few time windows in which cardiomyocytes can proliferate in preadolescent mouse hearts [1]. Indeed, the cardiomyocytes population expansion was observed during postnatal days 1–4 (P1–4) and days 14–18 (P14–18), with a 40% increase in cardiac cell number during each period. Interestingly, it is possible to achieve 100% regeneration in mouse hearts exposed to myocardial infarction at P1–4, but only 30% when treatment is delayed until P14–18, suggesting this proliferative burst actually improves reparative capability to counteract and compensate for heart injuries created in a mouse model [2]. Further fate mapping studies have also revealed that both extra- and intracellular mechanisms contribute to this temporary enhancement in proliferative ability. This review aims to highlight the most recent findings illustrating the causal mechanism links between cardiac regenerative signals and burst of cardiomyocyte proliferation.

## 2. Extracellular Signals

Myocardial extracellular space provides a microenvironment for tissue regeneration wherein cardiogenesis can be directly differentiated from cardiac progenitors and induced by a variety of secretory factors. It was reported recently that triiodothyronine (T3), neuregulin-1 (NRG1), follistatin-like 1 (Fstl1), and TWEAK regulate cardiogenesis and cardiac regeneration in a temporally or spatially dependent pattern (Table 1).

### 2.1. Triiodothyronine

**Figure 1:**
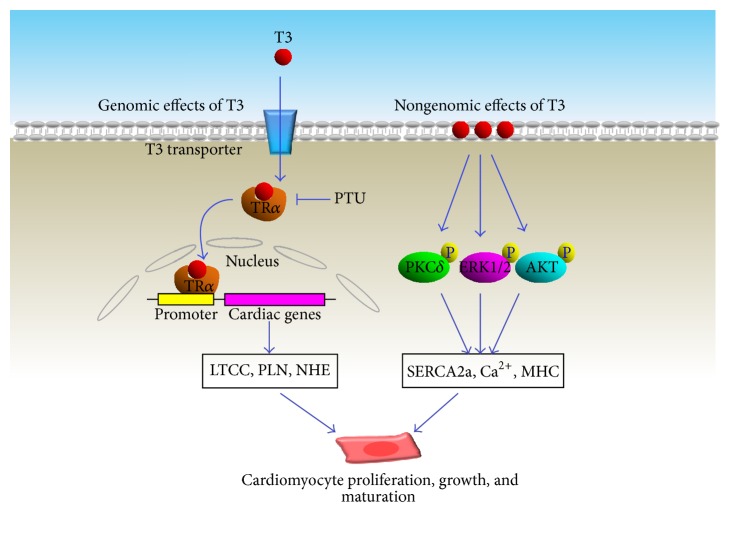
T3 promotes the proliferation, growth, and maturation of cardiomyocytes. LTCC: L-type calcium channel; PLN: phospholamban; NHE: Na^+^/H^+^ exchanger; MHC: myosin heavy chain.

Triiodothyronine (T3), a thyroid hormone derived from its prohormone thyroxine (T4) through biocatalysis induced by a thyroid-stimulating hormone, is released from pituitary gland. After it is released from the thyroid body, T3 is transported into the myocardium through circulation distribution. There are two spiked T3 concentrations that appear in mouse serum during preadolescent ages. The first of these occurs at birth and drops on postnatal day 7 (P7), while another T3 peak value appears between P10 and P12 causing a 5.6-fold increase compared to basal conditions. Interestingly, pharmacological inhibition of T3 biosynthesis using propylthiouracil (PTU) significantly suppressed cardiac growth during P14-P15, a crucial period during which the population of mononucleated cells increases 2.6-fold associated with a 36-fold increase in mitosis events in the left ventricle [2]. These results indicate that T3 promotes both cardiac growth and cardiomyocyte proliferation. Studies previously revealed that T3 can promote cardiomyocyte cell-cycle withdrawal [3] and enhance cardiomyocyte maturation with downregulation of cell proliferation [4].* In vitro* experiments in fetal sheep have indicated that the population of terminally differentiated multinucleated cells expands in association with enlarged cardiomyocyte size after T3 treatment [3]. Correspondingly, T3-dependent alterations in cardiomyocyte maturation are accompanied by changes in expression of contractility-regulating proteins, heart pacemaker, and calcium handling proteins. Particularly, abundance and maturation of multiple adrenergic receptors in differentiated cardiomyocytes are considered to be tuned up by the presence of T3. Although the molecular mechanism has not been elucidated for T3-induced cardiomyocyte proliferation, the signaling pathways that account for T3-induced cardiomyocyte maturation have been extensively studied. Thyroid hormone receptor *α* (TR*α*), a nuclear hormone receptor, has been demonstrated as the major protein molecule that receives T3 signaling. Upon T3 stimulation, TR*α* can bind to distal or proximal promoter elements to regulate cardiac gene expressions during myocardial growth. Indeed, TR*α* activation increased transcriptional activity of promoters, which subsequently regulate gene expressions of Na^+^-H^+^ exchanger (NHE) [5], voltage-gated potassium channel, L-type calcium channel [6], and phospholamban [7]. A recent study revealed that T3-induced cardiomyocyte growth can be abrogated by muscle ring finger-1 (MuRF-1), a muscle-specific ubiquitin ligase [8]. Such a noncanonical ubiquitination mechanism, along with the process of SUMOylation [9], posttranscriptionally modified the lysine residues and conformationally altered the ligand-binding domain (E/F) region of TR*α*, consequently inhibiting T3-induced TR*α* activation. Nongenomic effect of T3 was also revealed in adult cardiomyocyte, as evidenced by the rapid increase in phosphorylation of several kinases, AMPK [10], ERK1/2 [11], PKCdelta, p38-MAPK, and AKT, associated with the upregulation of sarcoplasmic reticulum Ca^2+^-ATPase (SERCA) and *α* and *β*-myosin heavy chain (MHC). Interestingly, this activation can be inhibited when T3's binding to cell membrane was pharmacologically antagonized, indicating that this TH effect is mediated through a cell membrane-initiated mechanism [12] (Figure 1). A current study also claims that T3 coordinates with the IGF-1 signaling pathway to mediate these cardiogenesis and maturation effects, which are responsible for the key structural and functional changes of the postnatal heart [13, 14].

Intriguingly, T3/TR*α* is also involved in cardiogenesis and cardioprotection in the presence of ischemic injury [15–17]. In an experimental model of acute myocardial infarction, T3 treatment has been shown to improve heart performance while decreasing cardiac remodeling [16, 18, 19], favorably improving cardiomyocyte shape and the geometry of left ventricular cavity [20]. The important role of T3 in maintaining heart performance has also been demonstrated by clinical investigation. Indeed, serum concentration of T3 was significantly reduced in patients with idiopathic dilated cardiomyopathy [21], and the mortality was significantly increased in cardiac disease patients with thyroid dysfunction [22]. These clinical data indicated that administration of T3 may provide a therapeutic opportunity for heart failure and myocardial repair. Actually, beneficial effects of short-term synthetic L-T3 replacement therapy have been revealed in dilated cardiomyopathy patients with low T3 syndrome, as evidenced by the remarkable improvements in heart remodeling, the enhanced resting cardiac output, and the reduced systemic vascular resistance [23, 24]. However, these therapeutic effects were not displayed when long-term L-T3 treatment was performed on chronic and stable heart failure patients with low serum T3 level [25]. Such an ineffective response might be therapeutic timing in relation to the course of the disease. Moreover, undesirable outcomes, such as arrhythmias, myocardial ischemia, or hemodynamic instability may be encountered during T3 therapy, and an excess of triiodothyronine administration may be associated with weight loss, increased heart rate, fatigue, reduction in serum cholesterol, and suppressed TSH- all signs and symptoms associated with thyrotoxicosis.

### 2.2. Neuregulin-1 (NRG1)

Neuregulin-1 is a 44-KD glycoprotein that serves as a direct ligand for ERBB tyrosine kinase receptors resulting in increased phosphorylation on tyrosine residues. In past few years, the major progress has been made in understanding the biological functional role of NRG1-ErbB axis in the regulation of neurodevelopment, synaptic plasticity, and synaptic transmission [26]. Indeed, NRG1 has shown the essential role in controlling rapid impulse conduction in the central nervous system through determining the myelination of an individual axon [27], and the disorders in NRG1-ErbB signaling have been etiologically implicated in schizophrenia [28], Parkinson's disease [29], Alzheimer's disease [30], and Hirschsprung disease [31]. Importantly, administration of NRG1 displayed protective effects in experimental model of neuron injury [32], and this trophic factor can significantly attenuate cognitive function and improve behavioral performance [33].

**Figure 2:**
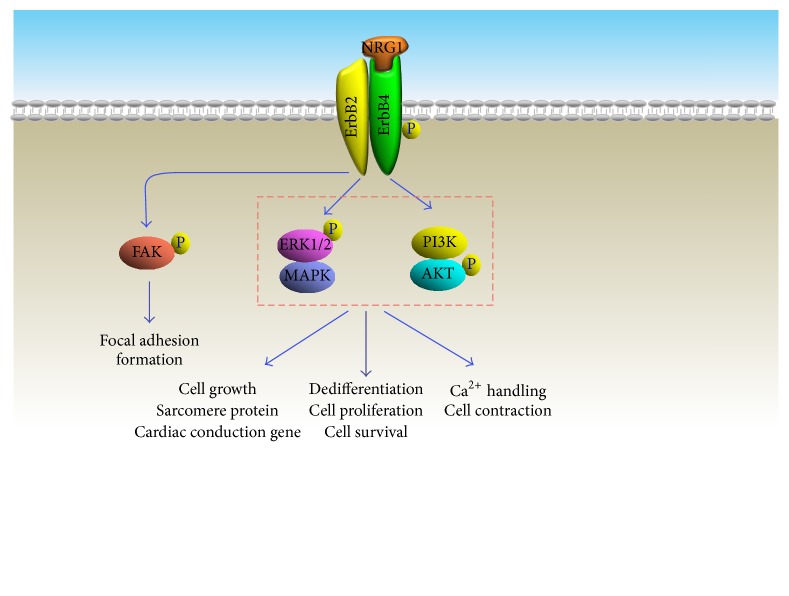
Signal pathways contributing to NRG1-induced cardiac regenerative effects.

It is well accepted that a variety of different isoforms can be generated from the NRG1 gene by alternative splicing process. There are fifteen NRG1 isoforms that have been identified so far, and these isoforms are distinguished based on their N-terminal sequence, receptor affinity-determined region (EGF-like domain), and a membrane-associated synthetic site. Upon proteolytic cleavage by transmembrane proteases, neuregulin-1 is activated and released, and EGF-like domains will then bind covalently with ErbB receptors. Particularly, the binding of neuregulin-1 to the extracellular ligand-binding domain of ErbB receptors induces a structural conformational change and subsequently results in the homodimerization of these receptors, by which ErbB receptor intracellular kinase domains phosphorylate their dimerization partner's C-terminus. These alterations start serial downstream signaling pathways, including ERK/MAPK [34], PI3K/AKT [35, 36], and FAK [37], which are responsible for heart development, cardiac structure maintenance [38], and functional integrity of heart muscle [39–41] (Figure 2).

The importance of NRG1-ErbB signaling in regulating cardiac development has been researched primarily using cardiomyocyte-specific ErbB gene knockout mouse, conditional ErbB transgenic mouse, and zebrafish models [42, 43]. The formation of ventricular trabeculations and atrioventricular cushions stagnated in ErbB knockout mice, leading to death during midembryogenesis. This similar lethal phenotype also occurred in NRG1-deleted mouse during embryogenesis. Although the severe fatal phenotype was not observed in ErbB deficient zebrafish, myofibril disarrangement jeopardized structural maintenance by altering the spatiotemporal organization of cardiomyocytes [44]. Developmental biological evidence revealed that trabeculation was delayed in ErbB deficient zebrafish, associated with the decreased heart contractile function. In addition protein synthesis, F-actin organization, and physiological hypertrophic responses were reinforced in rat neonatal cardiomyocytes after exposure to NRG1, while pretreatment with rapamycin blocked these effects, suggesting that activation of phosphatidylinositol 3 kinase (PI3K)/p70S6K signal cascade contributes to NRG1-induced myosin protein synthesis and fibril organization.

Interestingly, NRG1-ErbB signaling has also been implicated as having a key role in regulation of cardiac conduction system development. An initial study using an* in vivo* gene reporter system demonstrated that NRG1 could convert murine contractile cardiomyocytes isolated from 9.5-day postcotium embryos into conduction cells, and expression of these conduction system genes increased in a dose-dependent pattern [45]. A further study indicated that NRG1 treated hearts demonstrate enhanced electrical conduction and integration based on evidence that cardiac impulses propagated from the atrioventricular (AV) canal not only along the dorsal aspect of the ventricles, but also along the ventral aspect of the ventricle from the AV canal region [46]. Research on NRG1-ErbB signaling has contributed to stem cell therapeutics regulating cardiac development. When exposed to NRG1 on days 1–3 after differentiation, embryonic stem cells were more likely to differentiate into pacemaker cardiomyocytes. However, cells were more likely to differentiate into contractile cardiomyocytes when ESCs were treated NRG1 on days 5–9 after differentiation, suggesting that the temporal expression pattern of NRG1 is crucial in determining differentiation termination of heart cells. Unfortunately, these results somewhat contradict* in vivo* reports in which the cardiac conduction system developed only after the formation of heart trabeculation and compaction [47].

Regulation of cardiomyocyte proliferation during adult stages has received enormous attention in regenerative medical research, and some intriguing hints have been implied from comparative biological analysis among hearts from mammals, newts, and zebrafish. For instance, turnover of both mouse and human cardiomyocytes occurs at a rate of 1% per year, while the regeneration rate of adult cardiomyocytes can reach 30% in adult zebrafish heart within a few months of the injury. Importantly, NRG1-ErbB signaling has been identified as a mechanism contributing to cardiomyocyte proliferation and regeneration in zebrafish hearts [43], which is a tantalizing goal for those investigating this signal pathway on mammal hearts. D'Uva et al. provided experimental evidence that the ErbB receptor is required for cardiac cell growth in mice just after birth, and NRG1-induced cardiomyocyte proliferation diminished, owing to the reduced expression of ErbB, at 1 week after birth, the same time point as cardiomyocytes lose proliferation ability [42]. However, specific induction of ErbB in juvenile or adult cardiomyocytes extended the heart regenerative capacity into adulthood, restoring cardiac regenerative ability. In addition, the disassembled muscle contractile apparatus was observed in mature cardiomyocyte in response to overexpression of activated ErbB, indicating that ErbB can promote cardiomyocytes to partially dedifferentiate to a less-specialized phenotype, and this morphological alteration may facilitate cardiomyocyte proliferation in hearts of juvenile and adult mice.

In light of these findings, NRG1 treatment has been studied on several experimental heart injury models. Polizzotti et al. simulated myocardial infarction on the hearts of newborn mouse pups through localized freezing, and recombinant NRG1 was administrated to stimulate regeneration of heart muscle cells [48]. As predicted, heart tissue scarring and reduced heart function were exhibited in wild type hearts, whereas heart performance and cardiomyocyte proliferation were improved in NRG1 treated newborn mice. However, these beneficial effects did not emerge when NRG1 treatment was initiated later than 4 days after birth, suggesting that NRG1 treatment efficacy is largely dependent on the individual age of those receiving therapy. This limitation may be owing to the postnatal loss of ErbB expression [42], which has been correspondingly addressed in human heart regeneration studies. After isolation and purification of cells from biopsies of diseased human hearts, cardiomyocytes were cultured* in vitro* and then exposed to NRG1. The cardiomyocytes from heart biopsies of newborns displayed robust proliferation capacity after NRG1 treatment, but this effect was significantly reduced in cardiomyocyte biopsy samples obtained from 6-month-old hearts. Therefore, identification of suitable NRG1 therapeutic windows might improve the success rate for heart regenerative treatments and cardiac reconstructive surgeries. Clinical trials have been designed and performed to evaluate the efficacy and safety of recombinant human NRG1 (rhNRG-1), a 61 amino acid peptide, for treating stable chronic heart failure (CHF) since 2010 [49]. In phase I clinical trial, favorable acute and chronic hemodynamic effects were observed in patients with stable CHF after parenteral administration of rhNRG-1 [50]. Excitingly, a randomized, double-blind, placebo-controlled, and multicenter-performed phase II study, which is based on standard therapy, has demonstrated that rhNRG-1 can improve the cardiac function of patients with NYHA class II and III heart failure, as evidenced by the increased LVEF%, the reduced ESV, and EDV compared with pretreatment [51]. It has been authorized to perform phase III clinical trials in US by FDA at the end of 2013, and these promising clinical trial results support moving this program forward aggressively (http://www.zensunusa.com/clinicaltrials/index.aspx).

### 2.3. Follistatin-Like 1 (Fstl1)

Fstl1 is a secreted glycoprotein which was originally identified and cloned from mouse osteoblastic cell line. This protein is highly conserved among mouse, rat, and human, as reveled by more than 92% amino acid sequences identity. Although this follistatin-related protein is expressed predominantly in the cells of the mesenchymal lineage and widely involved in regulating development of several organs such as lung, ureter, and skeletal and central nervous system, recent studies have demonstrated this mesenchymal-derived factor also participates in immune response, carcinogenesis, and tumor metastasis through a paracrine manner [52].

An early study on AKT-inducible transgenic mouse indicated that Fstl1 protein and transcript expression are increased in hearts in response to AKT activation, and Fstl1 can be secreted from cardiomyocytes to produce cardioprotective effects through a paracrine mechanism [53]. This secretory protein is composed of 4 functional structural domains: (1) follistatin module containing 10 conserved cysteine residues; (2) an extracellular segment called Kazal-type serine protease inhibitor domain; (3) 2 EF-hand calcium-binding domains; (4) a Von Willebrand factor type-C domain. Interestingly, this multiple-functional secretory protein participates in a variety of physiological and pathological processes including heart remodeling, cardiogenic regulation, cardiomyocyte proliferation and division, and cardiac regeneration.

**Figure 3:**
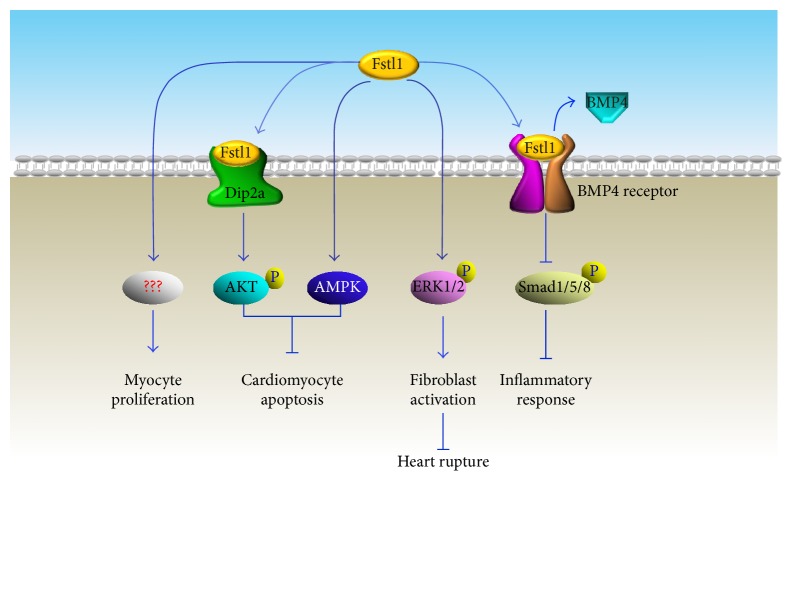
Fstl1-induced effects on cardiac regeneration and repair.

Enhanced Fstl1 expression in heart tissue and increased serum Fstl1 levels were observed in heart failure mouse models [54]. The experimental research on genetically modified mice demonstrated that neither Fstl1 ablation nor overexpression affected the heart size or contractile function under basal conditions, but the detrimental effects appeared in Fstl1 deficient mouse hearts in response to overload pressure. Specific disruption of cardiac* Fstl1* gene led to exacerbated cardiac hypertrophy and left ventricular dysfunction after thoracic aortic constriction (TAC) injury, indicating that Fstl1 functions as a negative regulator of cardiac growth under stress conditions. Interestingly, Fstl1 treatment attenuated epinephrine-induced cardiomyocyte hypertrophic response and protected against cardiac fibrosis in the presence of pathological hypertrophic stimuli. Coincidentally, the therapeutic impact of Fstl1 has also been observed in ischemia-reperfusion (I/R) injuries in both small and large preclinical models [55, 56]. Administration of Fstl1 protein significantly attenuated I/R-induced myocardial infarct areas associated with reduced apoptosis and decreased detrimental immune responses. Mechanistically, Fstl-1-induced cardiac protective effect is mediated by several independent signaling cascades. As a receptor of Fstl-1, expression of Dip2a (disconnected interacting protein 2 homolog A) has been identified on the cell surface of cardiomyocytes and endothelial cells. Along with AMPK activation-induced cell protective effects [57], upon binding with Fstl1, Dip2a can upregulate AKT phosphorylation to protect cell from hypoxia/reoxygenation-induced apoptosis and promote neovascularization by regulating endothelial cells' migration and differentiation [58]. During the early stage response to injury, Fstl1-induced ERK activation can activate fibroblasts to protect the heart from rupture and is considered as the essential cellular and molecular mechanisms for acute repair of the infarcted myocardium [55]. Despite that Fstl1-induced proinflammatory effects in immune cells have been demonstrated both* in vitro* and* in vivo* [52], detrimental myocardial inflammatory response was indeed alleviated in the presence of Fstl1. Such an opposite response may be attributed to the receptor competitive effects between Fstl1 and bone morphogenetic protein 4 (BMP4). Actually, Fstl1 can play as antagonist of bone morphogenetic protein receptor type II (BmpRII) and directly negatively regulate Smad1/5/8 phosphorylation [59], which consequently abolished BMP4-induced cell programmed death and BMP4-dependent induction of mediators [57] (Figure 3).

Most recent studies have identified Fstl1 as an epicardial cardiomyogenic factor. Enhanced mature cardiomyocyte features were observed when mouse embryonic stem cell- (ESC-) derived cardiomyocytes were cocultured with epicardial mesothelial cells (EMCs). Importantly, EMC-derived conditioned medium increased the number of beating colonies and promoted rhythmic calcium transients in ESC-derived cardiomyocytes. Interestingly, the incidence of aurora B kinase positive signals was doubled in ESC-derived cells, indicating that cell proliferation capacity was intensified after exposure to EMC-derived conditioned medium. Among 1596 mass spectrometry-recognized bioactive proteins in EMCs-derived conditioned medium, Fstl1 was identified as the major contributor to these cardiomyogenic and proliferative effects [56]. Finally, implantation of recombinant human Fstl1 via epicardial cell patch stimulated cell-cycle entry and promoted the division of preexisting cardiomyocytes, attenuating cardiac dysfunction and improving survival in mouse and porcine myocardial infarction models [56].

Despite showing promising therapeutic effects in preclinical experiments, it remains elusive whether the contributions of this protein are beneficial or detrimental to the development of human cardiovascular diseases. For instance, heart failure patients with increased Fstl1 levels in serum and the myocardium maintained the highest risk of mortality, suggesting that Fstl1 may serve as a biomarker in chronic systolic heart failure [60]. In addition, plasma Fstl1 levels were elevated in patients with acute Kawasaki disease, a major cause of acquired coronary aneurysm in childhood [61].

### 2.4. Tumor Necrosis Factor Ligand Superfamily Member 12 (TNFSF12)

TNFSF12, also called TNF-related weak inducer of apoptosis (TWEAK), is a multifunctional cytokine that is encoded by the* TNFSF12* gene. TWEAK is a type II transmembrane protein that belongs to the TNF ligand family. This protein is composed of four major domains, an N-terminal cytoplasmic domain, a transmembrane domain followed by an extracellular furin-identified stalk region, and the C-terminal tumor necrosis factor (TNF) homology domain (THD). Furin can function as an endoprotease that targets and cleaves TWEAK extracellular stalk peptide sequences, resulting in a soluble ligand containing the THD. Notably, both membrane-bound and soluble TWEAK (mTWEAK and sTWEAK) can be trimerized as a homogeneous complex to fully function and regulate similar cellular biological functions by binding to fibroblast growth factor-inducible 14 (Fn14) receptors, the smallest member of the tumor necrosis factor receptor (TNFR) superfamily. Specifically, site-mutagenesis studies have demonstrated that TWEAK can covalently bind to the single cysteine-rich domain (CRD) in the extracellular ligand-binding region of Fn14 receptor, which is determined by 3 evolutionarily conserved amino acid residues (ASP, Lys, and Met) in this domain [62]. Subsequently, a short cytoplasmic tail of Fn14 receptor will recruit TNFR-associated factor (TRAF) to regulate multiple cellular activities including apoptosis, migration, differentiation, proliferation, angiogenesis, and inflammation [63–66].

Activation of the TWEAK/Fn14 pathway in leukocyte subsets is thought to be a mediator of tissue response under pathological conditions. Activation of this pathway in nonhematopoietic tissue cell types also actively contributes to shaping of the tissue repair process, including the inflammatory response, tissue fibrosis, and functional components of regeneration [67]. Indeed,* in vivo* experimental evidence has shown that both TWEAK and Fn14 expression levels were significantly upregulated in remodeling cardiac tissue after myocardial infarction [68]. Further studies have also indicated that cardiomyocytes serve as a cell source reserve for the increased TWEAK and Fn14 cells under stressful conditions, an induction mediated by the Rho/ROCK pathway [68]. Although TWEAK/Fn14 activation can promote NF-*κ*B nuclear translocations that trigger inflammatory responses in injured heart tissue, proproliferative effects have also been observed in mammalian cardiomyocytes exposed to TWEAK treatment [69]. TWEAK can stimulate DNA synthesis in neonatal cardiomyocytes in a dosage-dependent manner associated with increased expression of proliferative markers cyclin D2 and Ki67, while reducing the amount of endogenous cell-cycle inhibitor p27/KIP. These studies demonstrated that TWEAK-induced cell-cycle reentry, mitosis, and cytokinesis are mediated by activating extracellular signal-regulated kinase (ERK) and phosphatidylinositol 3 kinase (PI3K). Conversely, TWEAK-induced proliferation was not detected in adult cardiomyocytes, which may be due to the decreased Fn14 expression during postnatal stage. This hypothesis was partially verified in isolated murine cardiomyocytes response to Fn14 inhibition. Indeed, TWEAK-induced neonatal cardiomyocytes DNA synthesis and proliferation were significantly inhibited when endogenous Fn14 was specifically blocked by a neutralizing antibody or siRNA. A recent study on cardiomyocytes using coimmunoprecipitation and proximity ligation assays revealed the interactions between endogenous Fn14 and fibroblast growth factor receptor-1 (FGFR-1), which serve an essential role in promoting cell proliferation. This interaction becomes more pronounced in the presence of TWEAK or FGF-1, and the synergistic interaction can stimulate cell-cycle reentry of >40% adult cardiomyocytes [70].

**Figure 4:**
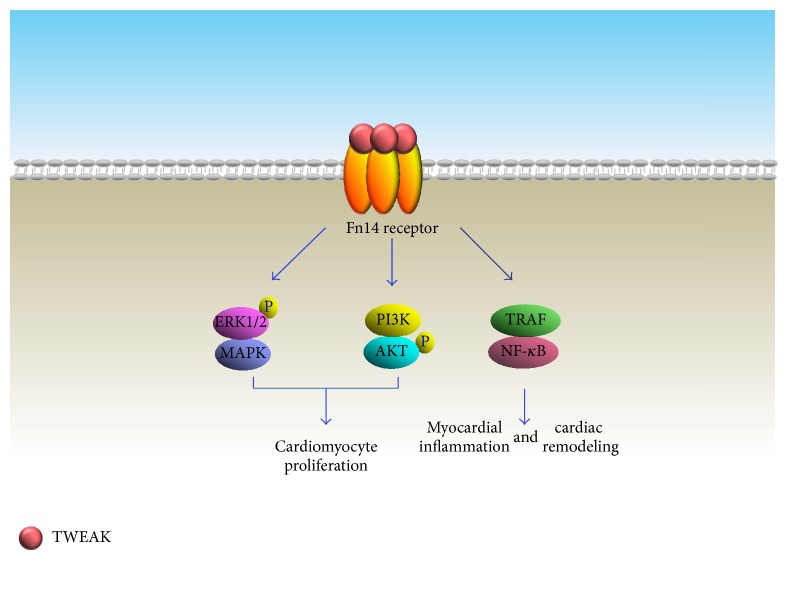
Involvement of TWEAK-Fn14 signaling pathways in cardiac regeneration.

Due to TWEAK/Fn14's modulatory effects in tissue response and repair, an attempt has been made to investigate whether TWEAK administration can provide therapeutic options for patients after myocardial infarction. Unfortunately, heart damage effects have been revealed in several preclinical experiments. Along with fibroblast proliferation and myofibroblast differentiation, Fn14 overexpression strengthened hypertrophic responses in cultured adult rat cardiomyocytes, which may lead to pathological remodeling in response to pressure overload stimulation [71, 72]. Correspondingly, treatments of mice with a recombinant human serum albumin conjugated-TWEAK induced myocardial healing defects after MI, associated with an exaggerated neutrophil infiltration into the myocardium [73]. In another study, systemic administration of TWEAK displayed maladaptive effects after MI, as revealed by worsened left ventricular function and the enhanced late mortality. Further molecular analysis revealed that expressions of PGC-1*α* and oxidative phosphorylation-regulating genes were significantly suppressed in cardiomyocytes, indicating such detrimental responses are exerted most likely via direct effects on cardiomyocytes [74] (Figure 4). TWEAK-induced maladaptive effects were partially supported by a clinical investigation that soluble TWEAK serum level was increased to a higher level in chronic heart failure with reduced ejection fraction (HF-REF) compared to healthy subjects [83]. Therefore, more preclinical* in vivo* experiments are necessary in order to elucidate the role of TWEAK in cardiac repair procedure.

## 3. Intracellular Signals

Signals within intracellular compartments not only participate in the regulation of cell bioactivities under physiological conditions, but also receive and transmit warning signals that coordinate stress responses and tissue repair after injury. Several intracellular signal pathways including PI3K-AKT, Hippo-Yap, cardiogenic transcription factors, and microRNAs have been identified as prominent regulators in processing cardiac development and regeneration.

### 3.1. PI3K-AKT Pathway

The PI3K-AKT pathway has been extensively studied and is recognized as a prominent intracellular signaling pathway in regulating a diverse selection of cellular functions and processes such as glucose uptake, energy metabolism, cell-cycle progression, apoptosis, and gene transcriptional regulation. Class 1A PI3K is a heterodimeric complex composed of a p110 catalytic subunit and a p85 regulatory subunit located on the plasma membrane. Upon activation by a variety of stimuli including growth factors, attachment of extracellular matrix, and oncogene products, PI3K can phosphorylate phosphatidylinositol 4,5-bisphosphate (IP2) into phosphatidylinositol 3,4,5-trisphosphate (IP3), which serves as a principle intracellular lipid second messenger to recruit downstream signals [84]. As an important downstream effector of PI3K, protein kinase B (PKB, also known as AKT) can employ its pleckstrin homology (PH) domain to bind IP3 and subsequently undergo a conformational change triggering PKB activation through 3-phosphoinositide-dependent protein kinase-1- (PDK1-) induced phosphorylation [85].

PI3K-AKT activation is required and linked to restriction point progression for G1-to-S transition, which is determined primarily by cyclin D-dependent kinase- (CDK-) induced phosphorylation of pRb. Cyclin D1, whose expression can be induced by c-Myc, can interact with CDK to promote pRb phosphorylation. Unfortunately, both cyclin D1 and c-Myc are proteins with short half-lives, because these signals are susceptible to proteolytic degradation when exposed to glycogen synthase kinase 3 beta (GSK-3*β*). Indeed, nuclear translocation of cyclin D1 will be blocked when GSK-3*β*-induced phosphorylation occurs at residue threonine-286 (T286), resulting in ubiquitin-mediated proteolytic degradation in the cytoplasm [86]. Constitutive expression of active PKB can prolong the half-life of cyclin D, while pharmacological inhibition of PI3K speeds up cyclin D1 degradation, indicating that PKB-mediated GSK-3*β* inhibition can stabilize cyclin D1 [87]. Additionally, PKB can also upregulate expression levels of cyclin D and c-Myc at both the transcriptional and translational levels. The positive cell-cycle regulatory effects of cyclin D can be counteracted by a group of inhibitory proteins including p15, p21, and p27^Kip1^. Among them, p27^Kip1^ is a principle inhibitor required for maintaining cell quiescence, whereas reduction is critical for cell-cycle reentry [88]. It has been established that the stability of p27 is regulated by the PI3K-AKT pathway, since activation of this pathway decreased both p27 expression level and the accumulation of p27 observed in cells as a response to AKT inhibition [89].

A well accepted study revealed the role of PI3K in regulating the efficiency of G2/M phase progression [90]. The number of neonatal cardiomyocytes in the mitotic and cytokinesis phase was increased 50~100%, associated with enhanced CDK7 expression, after activation of PI3K-AKT signals by C3orf58 (a kind of hypoxia), and AKT-induced stem cell factor (HASF). CDK7 is identified as a subunit of the general transcription factor IIH (TFIIH) and a member of intricate network of CDKs to promote mitosis and cell division, ensuring the genetic materials are accurately and equally segregated between two daughter cells [91]. Interestingly, it was recently reported that AKT activation could dramatically accelerate and amplify the transcriptional reprogramming of mouse cardiac fibroblasts, a process in which functional cardiomyocytes can be induced and generated from autologous fibroblasts. Spontaneous beating occurred in approximately 50% of reprogrammed fibroblasts after 3 weeks of induction in the group that was treated with AKT plus cardiac transcription factors. The result was more mature cardiomyocytes features, including enhanced polynucleation, cellular hypertrophic gene expression, and metabolic reprogramming in the reprogramed cells [92].

### 3.2. Hippo-Yap Pathway

The Hippo signaling complex is composed of a cluster of cytoplasm-located protein kinases and two major transcription factors associated with corresponding regulators. These pathway components are enriched with WW domains and their cognate proline-rich interacting motifs provide an efficient signaling mechanism to sense upstream input and start off the downstream output [93]. Briefly, mammalian sterile 20-like kinase (Mst) activation can be initiated when exposed to diverse stress signals such as extracellular matrix stiffness, mechanic stress, cytoskeletal rearrangement, contact inhibition, and anoxemia [94]. This signal activation can directly mediate mitochondrial function to affect energy metabolism. Subsequently, Mst can transduce this activation to large tumor suppressor kinase-1/2 (Lats1/2) to phosphorylate Yap. Spatial alteration of Yap, which serves as a transcriptional coactivator, is principally determined by the phosphorylations at serine residues 127 and 379. Accumulating* in vitro* experimental evidence has shown that Yap is subjected to cytoplasmic retention and ubiquitin-dependent degradation upon Lats kinase-induced phosphorylation, whereas unphosphorylated Yap will emerge in the nucleus. This intensified Yap signal can be observed in the nucleus when residue serine 127 is changed into Alanine (S127A), a mutation that can keep this residue from phosphorylation. Correspondingly, Yap-induced biological effects (including cell proliferation) are also enhanced to a greater extent in experimental S127A mutation Yap subjects when compared with those with wild type Yap. Nuclear-located Yap cannot identify or interact with DNA-binding domains* per se*. When combined with TAZ, this Hippo effector can act as a coactivator to modulate the DNA-binding activity of TEAD, a critical transcriptional factor that initiates proliferative and prosurvival gene progression programs. TEAD owns a C-terminal protein-binding domain, characterized by an immunoglobulin-like beta-sandwich fold with two extra helix-turn-helix inserts. This structure enabled TEAD to precisely recognize and covalently bind to the TEAD-binding domain of Yap [95]. Accordingly, Yap wraps around the globular structure of TEAD and forms extensive interactions via three highly conserved interfaces [96]. TEAD also contains an N-terminal TEA-domain, a DNA-binding module that can interact with canonical M-CAT elements to regulate target gene expression. M-CAT sequence motif (5′-TCATTCCT-3′) has been identified in several gene promoters and is a decisive DNA region for the regulation of cell growth, differentiation, and epithelial-mesenchymal transition. Notably, the enhanced protein-protein interaction between Yap and TEAD has been identified as a molecular mechanism contributing to oncogenesis and metastasis, especially hepatocellular carcinoma and gastric cancer [97, 98], and pharmacological blockade of the Yap-TEAD complex formation may foster the development of novel therapy strategies for inhibiting tumor growth [99].

The importance of Yap in regulating heart development has been documented in several studies using gain- and loss-of-function approaches. Slower heartbeat and a decreased number of cardiac Troponin-positive cardiomyocytes were observed, consequently resulting in embryonic death in inducible Yap gene mutant embryos. Although cardiac looping or chamber formation was not affected, deletion of Yap led to a significant reduction in ventricular myocyte number compared with the wild type littermates due to the diminished proliferation of cardiomyocytes. Correspondingly, forced expression of YapS112A (a Yap mutant form that is constitutively active and localized to the nucleus) significantly promoted the proliferation of cardiomyocytes in the hearts of transgenic embryos, and YapS112A transgenic mice displayed an abnormally thickened myocardium and expanded trabecular layer compared with those of Yap transgenic mice [100]. This similarly compromised cellular phenotype was also observed in Mst deficient embryonic bodies (Mst^−/−^ EBs), in which beating cell clumps disappeared and the expressions of cardiac progenitor markers such as Nkx2.5, Tbx5, Mesp1, Isl1, and Baf60c were significantly suppressed. Further studies have revealed that Mst is involved in cardiogenesis through regulation of noncanonical wnt ligands. Indeed, expression and secretion of several noncanonical Wnt ligands such as Wnt2, Wnt2b, and Wnt5a were reduced in Mst^−/−^ EBs, whereas canonical Wnt ligand gene expression was not affected [101]. Numerous studies have provided evidence to support that Yap is a nexus of multiple signaling pathways in governing cardiac growth and survival. Yap builds up interlink among the Hippo pathway, Wnt pathway, and the IGF pathway to regulate *β*-catenin signaling and precisely control cardiac development [100].

**Figure 5:**
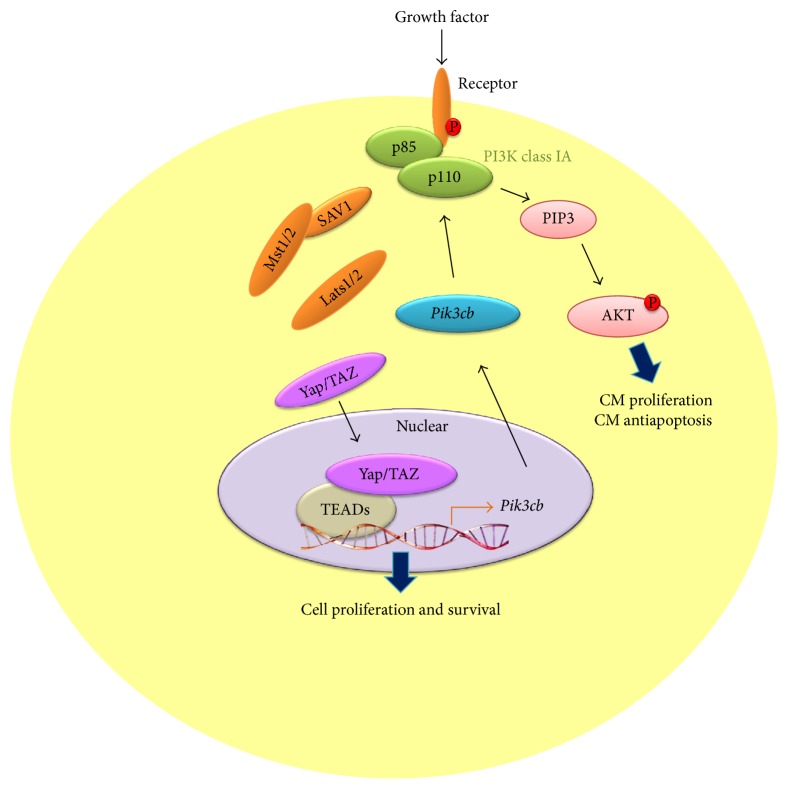
Pik3cb as a crucial direct target of Yap that links Hippo-Yap to PI3K-AKT signaling activation and regulates cardiomyocyte proliferation and survival. Dephosphorylated Yap/TAZ translocates into nuclear and directly activates target gene Pik3cb expression with several transcription factors (TEA-domain sequence-specific DNA-binding proteins). Pik3cb activation downstream of Yap promotes cardiomyocyte proliferation and survival by stimulating AKT activation.

In the process of cardiac differentiation, maturation of cardiomyocyte morphology is characterized by enhanced myofibril density and alignment, associated with visible sarcomeres under bright-field microscopy [102], while functional maturation is characterized by increased ion channel expression in the cell membrane, enhanced calcium storage capacity in the sarcoplasmic reticulum, high density distribution of adrenergic receptors, and robust contractility [103]. There is no evidence to date showing that the Hippo-Yap signal directly regulates the functional maturation of electrophysiology and calcium handling during cardiomyocyte differentiation. However, a most recent study implicated this signal pathway is involved in actin cytoskeletal remodeling with protrusion formation using the* Salvador* gene knockout (*Salv* KO) mouse model and chromatin immunoprecipitation sequencing (ChIP). Mst1 activation is dependent on the interaction with Salvador. Ablation of Salvador will inhibit the kinase activity of whole Hippo signals leading to accumulation of nonphosphorylated Yap in the nucleus. For example, Yap-Chip sequencing and mRNA expression profiling in* Salv* KO hearts revealed that Yap is involved in gene transcription and regulation of Sarcoglycan and Talin2, which composes the plasmalemmal complexes that link the actin cytoskeleton to the extracellular matrix. Importantly, this was confirmed in mouse ischemic hearts after left anterior descending artery ligation. The greater extent of cytoskeleton rearrangement was observed in Hippo kinase-compromised cardiomyocytes than in their wild type counterparts, enabling the migration of cardiomyocytes into the infarct border-zone. Upregulation of Sarcoglycan and Talin2 help* Salv* KO cardiomyocytes extends sarcomere-filled protrusions into scar tissue in the region of myocardial injury, as demonstrated by the appearance of costameres linking ECM to actin through the integrin-vinculin-talin complex, an essential cellular event for heart regeneration [104]. Interestingly, recent study demonstrated that Hippo-Yap function coordinates with PI3K-AKT pathway to promote cardiomyocyte proliferation and survival. Indeed, the p110 catalytic subunit of PI3K is encoded by gene,* Pik3cb*, which is the direct target of Yap. Through cooperation with its transcriptional partner TEAD, Yap can enhance* Pik3cb* expression, which subsequently induces AKT activation [105] (Figure 5).

### 3.3. Cardiogenic Transcription Factors

Cardiac transcription factors are protein molecules that can bind to specific DNA domains, such as promoters and enhancers, to specifically regulate expressions of cardiac genes. The biological functions of several cardiac transcription factors have been revealed in human congenital heart defects and confirmed in transgenic mice expressing dominant-negative mutant alleles. Also, downstream targets of these transcription factors have been demonstrated using next-generation RNA sequencing techniques. This part will highlight recent discovery in three cardiogenic transcription factors, especially their potentials for heart regeneration (Table 2).

#### 3.3.1. T-Box Transcription Factor 20 (TBX20)

The relevance of the TBX20 gene in maintaining heart development has been revealed in various forms of congenital heart diseases and in many animal models [106]. This gene encodes transcription factor TBX20, which is essential for development of the interatrial septum. A septal defect has also been observed in TBX20 mutants, resulting in deoxygenated blood flowing into the left atrium and left ventricle. TBX20 is characterized by the presence of a 180-residue DNA-binding domain (T-box), a highly conservative *β*-sheet structure among T-box protein family members. A palindromic consensus sequence 5′-T(G/C)ACACCTAGGTGTGAAATT-3′ has been identified using site-selection experiments, named the T-site, that has been recognized as T-box-targeted DNA region, and the half site of this segment 5′-AGGTGTGA-3′ (also named as T/2 site) was identified as a core motif that can efficiently and sufficiently interact with transcription factors [107].

Increased TBX20 gene expression was observed in endomyocardial biopsies from idiopathic dilated cardiomyopathy patients and animal models of cardiomyopathy that are associated with compromised heart performance [108], and upregulation of this gene is considered an adaptive response of a stressed heart. In the loss-of-function experiments, chamber dilation was observed in TBX20 knockout mouse hearts, and cardiomyocyte-specific ablation of this transcription factor resulted in animal lethality within 15 days [75]. Conversely, cardiac-specific overexpression of TBX20 significantly improved animal survival, increased heart contractile function, and reduced myocardial infarction after ischemia injury [76]. Genome-wide ChIP analysis of the TBX20-binding motif in the adult mouse heart indicated that many cardiac genes meant for encoding critical transcription factors, ion channels, and cytoskeletal/myofibrillar proteins are direct downstream targets of TBX20 [75]. Correspondingly, prolonged QRS duration and heart blockage were detected in TBX20-deficient mice, and electrophysiological disorders also appeared in isolated TBX20-null cardiomyocytes as evidenced by decreased levels of L-type calcium current, inactivation of potassium current, and compromised calcium cycling [75]. Ongoing studies have revealed that TBX20 overexpression can activate multiple cell proliferation signaling pathways (including PI3K-AKT, Hippo-Yap, and BMP/Smad1/5/8) in the myocardium of adult mice, while repressing cell-cycle inhibitory genes P21 and Meis1 to induce cardiomyocyte proliferation under both basal and detrimental stress conditions [76].

#### 3.3.2. GATA4

GATA4 is a member of the GATA protein family, which are transcription factors evolutionarily conserved in the context of structure and function. At the structural level, GATA4 is characterized by two highly conserved type IV zinc fingers located at the N-terminal and C-terminal. These two functional domains coordinate the specific binding of GATA4 to the A/TGATAA/G conserved region inside the DNA. Specifically, C-terminal zinc finger (along with the adjacent domain) is adequate and essential for specific covalent binding, while the N-terminal counterpart functions as an equalizer in stabilizing the protein-DNA interaction [109]. A recent report demonstrated that GATA4 transcriptional activity is mediated by cGMP-PKG signaling [77]. GATA4 is preferentially expressed in heart tissue, and the binding motifs have been identified within the promoter of genes that regulate cardiogenic differentiation, cell proliferation, and cell fate determination. A previous investigation revealed that a heterozygous G296S missense mutation of GATA4 is associated with an increased incidence of atrial and ventricular septal defects and pulmonary valve stenosis in humans [110]. In line with these findings, GATA4-deficient mice displayed embryonic lethality, a thin myocardium with reduced cardiomyocyte proliferation, and atrioventricular septum defects [111], and GATA-4 inactivation caused severe cardiac dysfunction [80]. Cyclin-dependent kinases (CDKs), especially CDK2 and CDK4, are the direct targets of GATA4. Dysfunction of GATA4 leads to the reduction of these CDKs and contributes to cardiac intraseptal defects in humans [78]. In addition, GATA4 can also promote cardiomyocyte replication through an indirect paracrine mechanism. A recent study demonstrated that a conserved region of the Fgf16 second intron can be identified and bound by GATA4, and the activation of this enhancer domain led to an increased expression and secretion of FGF16, which subsequently promoted cardiomyocyte proliferation and cardiac regeneration [79].

#### 3.3.3. NF-*κ*B

NF-*κ*B is a well-known transcription factor that regulates a variety of cellular biological effects including inflammation, cell proliferation, cell survival, and tissue growth. Under resting conditions, NF-*κ*B is sequestered and insulated by I*κ*B within the cytoplasm. Under stressful stimuli, proteasome-induced degradation of I*κ*B releases NF-*κ*B to induce gene expression after nuclear translocation. In the event of pressure overload, NF-*κ*B activation increases cardiac hypertrophic response, characterized by increased cardiomyocyte size and enhanced expression of fetal genes [81]. It was previously reported that cardiomyocyte-specific NF-*κ*B activation (which is induced by I*κ*B kinase (IKK) overexpression) can induce myocarditis, inflammatory dilated cardiomyopathy, and muscle fiber atrophy with robust interferon type 1 (IFN-1) responses resulting in heart failure [112]. However, a recent study using the zebrafish model system indicated that myocardial NF-*κ*B activation is essential for heart regeneration. The pronounced spatiotemporal correlation between NF-*κ*B activity and heart tissue regeneration was exhibited following injury, while myocardial blockade of NF-*κ*B activity suppressed cardiac regeneration with pleiotropic effects, including reduced cardiomyocyte proliferation and a blunted epicardial injury response. Interestingly, the signals of NF-*κ*B activation colocalize with GATA4 activation during the myocardial regenerative process, and GATA4-induced regulatory sequences can be prevented when NF-*κ*B signaling is antagonized, indicating that NF-*κ*B is an important signal for triggering a heart regeneration program. These studies imply that NF-*κ*B acts as a crucial node between cardiac injury and tissue regeneration, and precise spatiotemporal regulation of NF-*κ*B activity is an issue that determines the quality of heart tissue repair [82].

## 4. Conclusion and Perspective

Intracellular and extracellular molecular mechanisms both contribute to the complicated repair and renewal process of cardiac regeneration. Although textbooks suggest that the lost or damaged tissue cannot be replaced by adult mammalian heart regeneration, several studies have described evidence that neonatal mammalian hearts have some regenerative capacity, though it is lost beyond 7 days of age. This capability has encouraged the search for alternate effective ways to stimulate cardiac regenerative signal pathways promoting cardiac myocyte proliferation and differentiation. Current findings on cardiac regenerative signals will thus likely provide new therapeutic opportunities for the treatment of heart failure.

## Figures and Tables

**Table 1:** Extracellular signals for cardiac regeneration.

Extracellular signals	Receptor	Signal pathway	Biological effects		Clinical effects on HF patients
			*In vitro*	*In vivo*	
Triiodothyronine (T3)	TR1*α* (thyroid hormone receptor *α*1)	① AMPK↑ [10] ② Regulating IGF1/IGF1-R/AKT [13] ③ ERK↑ [11]	CM growth↑ [10], CM maturation↑ [4], myofilament proteins expression↑ [12], L-type Ca^2+^ channel↓ [6]	CM proliferation and cardiac regeneration↑ [2], cardiac ischemia-reperfusion injury↓ [15–17], cardiac remodeling↓ [16, 18, 19]	Ventricular performance↑ [23], cardiac index↑ [24], cardiac function not improved [25],

Neuregulin-1 (NRG1)	ErbB receptor	① ERK1/2→MAPK↑ [34] ② PI3K→AKT↑ [35, 36] ③ FAK↑ [37]	CM survival↑ [36], calcium handling↑ [39], CM communication↑ [40, 41], myofibrillar structural damage in response to ErbB inhibition [38]	Development of cardiac conduction system↑ [45, 46], CM dedifferentiation and proliferation↑ [42, 43], myofilament organization↑ [44]	cardiac output↑, LVEF↑, systemic vascular resistance↓, ESV↓ EDV↓ [50, 51]

Follistatin-like 1 (Fstl1)	DIP2A (disconnected interacting protein 2 homolog A)	① AMPK↑ [57] ② p-AKT↑ [58] ③ ERK1/2↑ [55]	Cell apoptosis↓ [58]	Cardiac rupture↓ [55], CM proliferation↑, heart performance↑ [56]	N/A

TNF-related weak inducer of apoptosis (TWEAK)	Fn14 receptor	① TRAF→NF-*κ*B↑ [68] ② ERK [70] ③ PI3K→AKT↑ [70]	CM cell cycle reentry↑ [70], cardiomyocyte proliferation↑ [69], CM-derived proinflammatory cytokines↑ [68], fibroblast proliferation and myofibroblast differentiation↑ [71]	Cardiac hypertrophy↑ [72], myocardial inflammatory responses↑ [73], heart performance↓ [74]	N/A

CM: cardiomyocyte; ↑: increase or intensify; ↓: decrease; LVEF: left ventricular ejection fraction; ESV: end-systolic volume; EDV: end-diastolic volume; N/A: not available.

**Table 2:** Cardiogenic transcription factor.

Cardiogenic transcription factor	Functional domain	Upstream signal	Direct target (gene)	Cardiac phenotype	
				*Knockout*	*Knock-in*
TBX20	T-box (*β*-sheet structure)	BMP/Smad	Ion channels, cytoskeletal proteins; myofibrillar proteins [75]	Ventricular dilation, long QRS wave, heart arrest [75],	Heart performance↑, CM proliferation↑ [76],

GATA4	Zinc fingers (type IV)	PKG-1*α* [77]	Cyclin-dependent kinases [78] FGF16 [79]	embryonic lethality [78], severe cardiac dysfunction [80]	heart function↑, cardiomyocyte replication↑, heart regeneration↑ [79]

NF-*κ*B	Rel homology domain (RHD)	N/A	Cardiac hypertrophic genes ANF, *β*-MHC↑ [81], GATA4 [82]	Zebrafish heart regeneration↓ [82]	N/A

↑: increase or intensify; ↓: decrease.

## References

[1] Palpant N. J., Murry C. E. (2014). Proliferation at the heart of preadolescence. *Cell*.

[2] Naqvi N., Li M., Calvert J. W. (2014). A proliferative burst during preadolescence establishes the final cardiomyocyte number. *Cell*.

[3] Chattergoon N. N., Giraud G. D., Louey S., Stork P., Fowden A. L., Thornburg K. L. (2012). Thyroid hormone drives fetal cardiomyocyte maturation. *The FASEB Journal*.

[4] Yang X., Rodriguez M., Pabon L. (2014). Tri-iodo-L-thyronine promotes the maturation of human cardiomyocytes-derived from induced pluripotent stem cells. *Journal of Molecular and Cellular Cardiology*.

[5] Li X., Misik A. J., Rieder C. V., Solaro R. J., Lowen A., Fliegel L. (2002). Thyroid hormone receptor *α*
_1_ regulates expression of the Na^+^/H^+^ exchanger (NHE1). *The Journal of Biological Chemistry*.

[6] Alonso H., Fernández-Ruocco J., Gallego M. (2015). Thyroid stimulating hormone directly modulates cardiac electrical activity. *Journal of Molecular and Cellular Cardiology*.

[7] Xie L., Pi X., Townley-Tilson W. H. D. (2015). PHD2/3-dependent hydroxylation tunes cardiac response to *β*-adrenergic stress via phospholamban. *The Journal of Clinical Investigation*.

[8] Wadosky K. M., Berthiaume J. M., Tang W. (2016). MuRF1 mono-ubiquitinates TR*α* to inhibit T3-induced cardiac hypertrophy in vivo. *Journal of Molecular Endocrinology*.

[9] Liu Y.-Y., Kogai T., Schultz J. J., Mody K., Brent G. A. (2012). Thyroid hormone receptor isoform-specific modification by small ubiquitin-like modifier (SUMO) modulates thyroid hormone-dependent gene regulation. *The Journal of Biological Chemistry*.

[10] Takano A. P. C., Diniz G. P., Barreto-Chaves M. L. M. (2013). AMPK signaling pathway is rapidly activated by T3 and regulates the cardiomyocyte growth. *Molecular and Cellular Endocrinology*.

[11] Pantos C., Xinaris C., Mourouzis I., Malliopoulou V., Kardami E., Cokkinos D. V. (2007). Thyroid hormone changes cardiomyocyte shape and geometry via ERK signaling pathway: potential therapeutic implications in reversing cardiac remodeling?. *Molecular and Cellular Biochemistry*.

[12] Iordanidou A., Hadzopoulou-Cladaras M., Lazou A. (2010). Non-genomic effects of thyroid hormone in adult cardiac myocytes: relevance to gene expression and cell growth. *Molecular and Cellular Biochemistry*.

[13] Chattergoon N. N., Louey S., Stork P. J., Giraud G. D., Thornburg K. L. (2014). Unexpected maturation of PI3K and MAPK-ERK signaling in fetal ovine cardiomyocytes. *American Journal of Physiology—Heart and Circulatory Physiology*.

[14] Forhead A. J., Fowden A. L. (2014). Thyroid hormones in fetal growth and prepartum maturation. *Journal of Endocrinology*.

[15] Forini F., Kusmic C., Nicolini G. (2014). Triiodothyronine prevents cardiac ischemia/reperfusion mitochondrial impairment and cell loss by regulating miR30a/p53 axis. *Endocrinology*.

[16] Pantos C., Mourouzis I., Saranteas T. (2011). Acute T3 treatment protects the heart against ischemia-reperfusion injury via TR*α*1 receptor. *Molecular and Cellular Biochemistry*.

[17] Mourouzis I., Kostakou E., Galanopoulos G., Mantzouratou P., Pantos C. (2013). Inhibition of thyroid hormone receptor *α*1 impairs post-ischemic cardiac performance after myocardial infarction in mice. *Molecular and Cellular Biochemistry*.

[18] Alonso-Merino E., Martín Orozco R., Ruíz-Llorente L. (2016). Thyroid hormones inhibit TGF-*β* signaling and attenuate fibrotic responses. *Proceedings of the National Academy of Sciences of the United States of America*.

[19] Zhang Y., Dedkov E. I., Lee B., Li Y., Pun K., Gerdes A. M. (2014). Thyroid hormone replacement therapy attenuates atrial remodeling and reduces atrial fibrillation inducibility in a rat myocardial infarction-heart failure model. *Journal of Cardiac Failure*.

[20] Pingitore A., Chen Y., Gerdes A. M., Iervasi G. (2012). Acute myocardial infarction and thyroid function: new pathophysiological and therapeutic perspectives. *Annals of Medicine*.

[21] Naderi N., Heidarali M., Barzegari F., Ghadrdoost B., Amin A., Taghavi S. (2015). Hormonal profile in patients with dilated cardiomyopathy. *Research in Cardiovascular Medicine*.

[22] Friberg L., Drvota V., Bjelak A. H., Eggertsen G., Ahnve S. (2001). Association between increased levels of reverse triiodothyronine and mortality after acute myocardial infarction. *American Journal of Medicine*.

[23] Pingitore A., Galli E., Barison A. (2008). Acute effects of triiodothyronine (T3) replacement therapy in patients with chronic heart failure and low-T3 syndrome: a randomized, placebo-controlled study. *The Journal of Clinical Endocrinology and Metabolism*.

[24] Goldman S., McCarren M., Morkin E. (2009). DITPA (3,5-diiodothyropropionic acid), a thyroid hormone analog to treat heart failure: phase II trial veterans affairs cooperative study. *Circulation*.

[25] Holmager P., Schmidt U., Mark P. (2015). Long-term L-Triiodothyronine (T3) treatment in stable systolic heart failure patients: a randomised, double-blind, cross-over, placebo-controlled intervention study. *Clinical Endocrinology*.

[26] Liu X., Bates R., Yin D.-M. (2011). Specific regulation of NRG1 isoform expression by neuronal activity. *The Journal of Neuroscience*.

[27] Lundgaard I., Luzhynskaya A., Stockley J. H. (2013). Neuregulin and BDNF induce a switch to NMDA receptor-dependent myelination by oligodendrocytes. *PLoS Biology*.

[28] Mostaid M. S., Lloyd D., Liberg B. (2016). Neuregulin-1 and schizophrenia in the genome-wide association study era. *Neuroscience & Biobehavioral Reviews*.

[29] Hama Y., Yabe I., Wakabayashi K. (2015). Level of plasma neuregulin-1 SMDF is reduced in patients with idiopathic Parkinson's disease. *Neuroscience Letters*.

[30] Chang K. A., Shin K. Y., Nam E. (2016). Plasma soluble neuregulin-1 as a diagnostic biomarker for Alzheimer's disease. *Neurochemistry International*.

[31] Gui H., Tang W. K., So M. T. (2013). RET and NRG1 interplay in Hirschsprung disease. *Human Genetics*.

[32] Guan Y.-F., Wu C.-Y., Fang Y.-Y. (2015). Neuregulin 1 protects against ischemic brain injury via ErbB4 receptors by increasing GABAergic transmission. *Neuroscience*.

[33] Engel M., Snikeris P., Jenner A., Karl T., Huang X.-F., Frank E. (2015). Neuregulin 1 prevents phencyclidine-induced behavioral impairments and disruptions to GABAergic signaling in mice. *The International Journal of Neuropsychopharmacology*.

[34] Chen M., Bi L.-L., Wang Z.-Q., Zhao F., Gan X.-D., Wang Y.-G. (2013). Time-dependent regulation of neuregulin-1*β*/ErbB/ERK pathways in cardiac differentiation of mouse embryonic stem cells. *Molecular and Cellular Biochemistry*.

[35] Pentassuglia L., Heim P., Lebboukh S., Morandi C., Xu L., Brink M. (2016). Neuregulin-1*β* promotes glucose uptake via PI3K/Akt in neonatal rat cardiomyocytes. *American Journal of Physiology—Endocrinology and Metabolism*.

[36] Jie B., Zhang X., Wu X., Xin Y., Liu Y., Guo Y. (2012). Neuregulin-1 suppresses cardiomyocyte apoptosis by activating PI3K/Akt and inhibiting mitochondrial permeability transition pore. *Molecular and Cellular Biochemistry*.

[37] Kuramochi Y., Guo X., Sawyer D. B. (2006). Neuregulin activates erbB2-dependent src/FAK signaling and cytoskeletal remodeling in isolated adult rat cardiac myocytes. *Journal of Molecular and Cellular Cardiology*.

[38] Pentassuglia L., Graf M., Lane H. (2009). Inhibition of ErbB2 by receptor tyrosine kinase inhibitors causes myofibrillar structural damage without cell death in adult rat cardiomyocytes. *Experimental Cell Research*.

[39] Brero A., Ramella R., Fitou A. (2010). Neuregulin-1*β*1 rapidly modulates nitric oxide synthesis and calcium handling in rat cardiomyocytes. *Cardiovascular Research*.

[40] Pentassuglia L., Sawyer D. B. (2013). ErbB/integrin signaling interactions in regulation of myocardial cell-cell and cell-matrix interactions. *Biochimica et Biophysica Acta—Molecular Cell Research*.

[41] McCormick M. E., Collins C., Makarewich C. A. (2014). Platelet endothelial cell adhesion molecule-1 mediates endothelial-cardiomyocyte communication and regulates cardiac function. *Journal of the American Heart Association*.

[42] D'Uva G., Aharonov A., Lauriola M. (2015). ERBB2 triggers mammalian heart regeneration by promoting cardiomyocyte dedifferentiation and proliferation. *Nature Cell Biology*.

[43] Gemberling M., Karra R., Dickson A. L., Poss K. D. (2015). Nrg1 is an injury-induced cardiomyocyte mitogen for the endogenous heart regeneration program in zebrafish. *eLife*.

[44] Reischauer S., Arnaout R., Ramadass R., Stainier D. Y. R. (2014). Actin binding GFP allows 4D in vivo imaging of myofilament dynamics in the zebrafish heart and the identification of Erbb2 signaling as a remodeling factor of myofibril architecture. *Circulation Research*.

[45] Rentschler S., Zander J., Meyers K. (2002). Neuregulin-1 promotes formation of the murine cardiac conduction system. *Proceedings of the National Academy of Sciences of the United States of America*.

[46] Tenin G., Clowes C., Wolton K. (2014). Erbb2 is required for cardiac atrial electrical activity during development. *PLoS ONE*.

[47] Rupert C. E., Coulombe K. L. K. (2015). The roles of neuregulin-1 in cardiac development, homeostasis, and disease. *Biomarker Insights*.

[48] Polizzotti B. D., Ganapathy B., Walsh S. (2015). Neuregulin stimulation of cardiomyocyte regeneration in mice and human myocardium reveals a therapeutic window. *Science Translational Medicine*.

[49] Cote G. M., Sawyer D. B., Chabner B. A. (2012). ERBB2 inhibition and heart failure. *The New England Journal of Medicine*.

[50] Jabbour A., Hayward C. S., Keogh A. M. (2011). Parenteral administration of recombinant human neuregulin-1 to patients with stable chronic heart failure produces favourable acute and chronic haemodynamic responses. *European Journal of Heart Failure*.

[51] Gao R., Zhang J., Cheng L. (2010). A phase II, randomized, double-blind, multicenter, based on standard therapy, placebo-controlled study of the efficacy and safety of recombinant human neuregulin-1 in patients with chronic heart failure. *Journal of the American College of Cardiology*.

[52] Chaly Y., Hostager B., Smith S., Hirsch R. (2014). Follistatin-like protein 1 and its role in inflammation and inflammatory diseases. *Immunologic Research*.

[53] Oshima Y., Ouchi N., Sato K., Izumiya Y., Pimentel D. R., Walsh K. (2008). Follistatin-like 1 is an Akt-regulated cardioprotective factor that is secreted by the heart. *Circulation*.

[54] Shimano M., Ouchi N., Nakamura K. (2011). Cardiac myocyte follistatin-like 1 functions to attenuate hypertrophy following pressure overload. *Proceedings of the National Academy of Sciences of the United States of America*.

[55] Maruyama S., Nakamura K., Papanicolaou K. N. (2016). Follistatin‐like 1 promotes cardiac fibroblast activation and protects the heart from rupture. *EMBO Molecular Medicine*.

[56] Wei K., Serpooshan V., Hurtado C. (2015). Epicardial FSTL1 reconstitution regenerates the adult mammalian heart. *Nature*.

[57] Ogura Y., Ouchi N., Ohashi K. (2012). Therapeutic impact of follistatin-like 1 on myocardial ischemic injury in preclinical models. *Circulation*.

[58] Ouchi N., Asaumi Y., Ohashi K. (2010). DIP2A functions as a FSTL1 receptor. *The Journal of Biological Chemistry*.

[59] Geng Y., Dong Y., Yu M. (2011). Follistatin-like 1 (Fstl1) is a bone morphogenetic protein (BMP) 4 signaling antagonist in controlling mouse lung development. *Proceedings of the National Academy of Sciences of the United States of America*.

[60] El-Armouche A., Ouchi N., Tanaka K. (2011). Follistatin-like 1 in chronic systolic heart failure: a marker of left ventricular remodeling. *Circulation: Heart Failure*.

[61] Gorelik M., Wilson D. C., Cloonan Y. K., Shulman S. T., Hirsch R. (2012). Plasma follistatin-like protein 1 is elevated in Kawasaki disease and may predict coronary artery aneurysm formation. *Journal of Pediatrics*.

[62] Dhruv H., Loftus J. C., Narang P. (2013). Structural basis and targeting of the interaction between fibroblast growth factor-inducible 14 and tumor necrosis factor-like weak inducer of apoptosis. *The Journal of Biological Chemistry*.

[63] Ensign S. P. F., Mathews I. T., Eschbacher J. M., Loftus J. C., Symons M. H., Tran N. L. (2013). The Src homology 3 domain-containing guanine nucleotide exchange factor is overexpressed in high-grade gliomas and promotes tumor necrosis factor-like weak inducer of apoptosis-fibroblast growth factor-inducible 14-induced cell migration and invasion via tumor necrosis factor receptor-associated factor 2. *The Journal of Biological Chemistry*.

[64] Girgenrath M., Weng S., Kostek C. A. (2006). TWEAK, via its receptor Fn14, is a novel regulator of mesenchymal progenitor cells and skeletal muscle regeneration. *The EMBO Journal*.

[65] Wiley S. R., Cassiano L., Lofton T. (2001). A novel TNF receptor family member binds TWEAK and is implicated in angiogenesis. *Immunity*.

[66] Moreno J. A., Sastre C., Madrigal-Matute J. (2013). HMGB1 expression and secretion are increased via TWEAK-Fn14 interaction in atherosclerotic plaques and cultured monocytes. *Arteriosclerosis, Thrombosis, and Vascular Biology*.

[67] Burkly L. C. (2015). Regulation of tissue responses: the TWEAK/Fn14 pathway and other TNF/TNFR superfamily members that activate non-canonical NF*κ*B signaling. *Frontiers in Immunology*.

[68] Chorianopoulos E., Heger T., Lutz M. (2010). FGF-inducible 14-kDa protein (Fn14) is regulated via the RhoA/ROCK kinase pathway in cardiomyocytes and mediates nuclear factor-*κ*B activation by TWEAK. *Basic Research in Cardiology*.

[69] Novoyatleva T., Diehl F., van Amerongen M. J. (2010). TWEAK is a positive regulator of cardiomyocyte proliferation. *Cardiovascular Research*.

[70] Novoyatleva T., Sajjad A., Pogoryelov D., Patra C., Schermuly R. T., Engel F. B. (2014). FGF1-mediated cardiomyocyte cell cycle reentry depends on the interaction of FGFR-1 and Fn14. *The FASEB Journal*.

[71] Novoyatleva T., Schymura Y., Janssen W. (2013). Deletion of Fn14 receptor protects from right heart fibrosis and dysfunction. *Basic Research in Cardiology*.

[72] Novoyatleva T., Janssen W., Wietelmann A., Schermuly R. T., Engel F. B. (2013). TWEAK/Fn14 axis is a positive regulator of cardiac hypertrophy. *Cytokine*.

[73] Pachel C., Mathes D., Bayer B. (2013). Exogenous administration of a recombinant variant of TWEAK impairs healing after myocardial infarction by aggravation of inflammation. *PLoS ONE*.

[74] Jarr K.-U., Eschricht S., Burkly L. C. (2014). TNF-like weak inducer of apoptosis aggravates left ventricular dysfunction after myocardial infarction in mice. *Mediators of Inflammation*.

[75] Shen T., Aneas I., Sakabe N. (2011). Tbx20 regulates a genetic program essential to adult mouse cardiomyocyte function. *The Journal of Clinical Investigation*.

[76] Xiang F.-L., Guo M., Yutzey K. E. (2016). Overexpression of Tbx20 in adult cardiomyocytes promotes proliferation and improves cardiac function after myocardial infarction. *Circulation*.

[77] Ma Y., Wang J., Yu Y., Schwartz R. J. (2016). PKG-1*α* mediates GATA4 transcriptional activity. *Cellular Signalling*.

[78] Misra C., Chang S.-W., Basu M., Huang N., Garg V. (2014). Disruption of myocardial Gata4 and Tbx5 results in defects in cardiomyocyte proliferation and atrioventricular septation. *Human Molecular Genetics*.

[79] Yu W., Huang X., Tian X. (2016). GATA4 regulates Fgf16 to promote heart repair after injury. *Development*.

[80] Prendiville T. W., Guo H., Lin Z. (2015). Novel roles of GATA4/6 in the postnatal heart identified through temporally controlled, cardiomyocyte-specific gene inactivation by adeno-associated virus delivery of Cre recombinase. *PLoS ONE*.

[81] Usui S., Maejima Y., Pain J. (2011). Endogenous muscle atrophy f-box mediates pressure overload-induced cardiac hypertrophy through regulation of nuclear factor-*κ*B. *Circulation Research*.

[82] Karra R., Knecht A. K., Kikuchi K., Poss K. D. (2015). Myocardial NF-*κ*B activation is essential for zebrafish heart regeneration. *Proceedings of the National Academy of Sciences of the United States of America*.

[83] Ptaszynska-Kopczynska K., Marcinkiewicz-Siemion M., Lisowska A. (2016). Alterations of soluble TWEAK and CD163 concentrations in patients with chronic heart failure. *Cytokine*.

[84] Okkenhaug K. (2013). Signaling by the phosphoinositide 3-kinase family in immune cells. *Annual Review of Immunology*.

[85] Dorn G. W., Force T. (2005). Protein kinase cascades in the regulation of cardiac hypertrophy. *The Journal of Clinical Investigation*.

[86] Ichikawa M., Sowa Y., Iizumi Y., Aono Y., Sakai T. (2015). Resibufogenin induces G1-phase arrest through the proteasomal degradation of cyclin D1 in human malignant tumor cells. *PLoS ONE*.

[87] Parekh P., Motiwale L., Naik N., Rao K. V. K. (2011). Downregulation of cyclin D1 is associated with decreased levels of p38 MAP kinases, Akt/PKB and Pak1 during chemopreventive effects of resveratrol in liver cancer cells. *Experimental and Toxicologic Pathology*.

[88] Chang F., Lee J. T., Navolanic P. M. (2003). Involvement of PI3K/Akt pathway in cell cycle progression, apoptosis, and neoplastic transformation: a target for cancer chemotherapy. *Leukemia*.

[89] Brazil D. P., Yang Z.-Z., Hemmings B. A. (2004). Advances in protein kinase B signalling: AKTion on multiple fronts. *Trends in Biochemical Sciences*.

[90] Maddika S., Ande S. R., Wiechec E., Hansen L. L., Wesselborg S., Los M. (2008). Akt-mediated phosphorylation of CDK2 regulates its dual role in cell cycle progression and apoptosis. *Journal of Cell Science*.

[91] Beigi F., Schmeckpeper J., Pow-Anpongkul P. (2013). C3orf58, a novel paracrine protein, stimulates cardiomyocyte cell-cycle progression through the PI3K-AKT-CDK7 pathway. *Circulation Research*.

[92] Zhou H., Dickson M. E., Kim M. S., Bassel-Duby R., Olson E. N. (2015). Akt1/protein kinase B enhances transcriptional reprogramming of fibroblasts to functional cardiomyocytes. *Proceedings of the National Academy of Sciences of the United States of America*.

[93] Salah Z., Aqeilan R. I. (2011). WW domain interactions regulate the Hippo tumor suppressor pathway. *Cell Death & Disease*.

[94] Zhou X., Wang Z., Huang W., Lei Q.-Y. (2014). G protein-coupled receptors: bridging the gap from the extracellular signals to the Hippo pathway. *Acta Biochimica et Biophysica Sinica*.

[95] Tian W., Yu J., Tomchick D. R., Pan D., Luo X. (2010). Structural and functional analysis of the YAP-binding domain of human TEAD2. *Proceedings of the National Academy of Sciences of the United States of America*.

[96] Li Z., Zhao B., Wang P. (2010). Structural insights into the YAP and TEAD complex. *Genes & Development*.

[97] Lamar J. M., Stern P., Liu H., Schindler J. W., Jiang Z.-G., Hynes R. O. (2012). The Hippo pathway target, YAP, promotes metastasis through its TEAD-interaction domain. *Proceedings of the National Academy of Sciences of the United States of America*.

[98] Qiao Y., Lin S. J., Chen Y. (2016). RUNX3 is a novel negative regulator of oncogenic TEAD–YAP complex in gastric cancer. *Oncogene*.

[99] Zhou Z., Hu T., Xu Z. (2015). Targeting Hippo pathway by specific interruption of YAP-TEAD interaction using cyclic YAP-like peptides. *The FASEB Journal*.

[100] Xin M., Kim Y., Sutherland L. B. (2011). Regulation of insulin-like growth factor signaling by Yap governs cardiomyocyte proliferation and embryonic heart size. *Science Signaling*.

[101] Li P., Chen Y., Mak K. K., Wong C. K., Wang C. C., Yuan P. (2013). Functional role of MsT1/MsT2 in embryonic stem cell differentiation. *PLoS ONE*.

[102] Lundy S. D., Zhu W.-Z., Regnier M., Laflamme M. A. (2013). Structural and functional maturation of cardiomyocytes derived from human pluripotent stem cells. *Stem Cells and Development*.

[103] Robertson C., Tran D. D., George S. C. (2013). Concise review: maturation phases of human pluripotent stem cell-derived cardiomyocytes. *Stem Cells*.

[104] Morikawa Y., Zhang M., Heallen T. (2015). Actin cytoskeletal remodeling with protrusion formation is essential for heart regeneration in Hippo-deficient mice. *Science Signaling*.

[105] Lin Z., Zhou P., von Gise A. (2015). Pi3kcb links Hippo-YAP and PI3K-AKT signaling pathways to promote cardiomyocyte proliferation and survival. *Circulation Research*.

[106] Monroy-Muñoz I. E., Pérez-Hernández N., Rodríguez-Pérez J. M. (2015). *Novel mutations* in the transcriptional activator domain of the human TBX20 in patients with atrial septal defect. *BioMed Research International*.

[107] Macindoe I., Glockner L., Vukašin P. (2009). Conformational stability and DNA binding specificity of the cardiac T-box transcription factor Tbx20. *Journal of Molecular Biology*.

[108] Mittal A., Sharma R., Prasad R., Bahl A., Khullar M. (2016). Role of cardiac TBX20 in dilated cardiomyopathy. *Molecular and Cellular Biochemistry*.

[109] El-Hachem N., Nemer G. (2011). Identification of new GATA4-small molecule inhibitors by structure-based virtual screening. *Bioorganic & Medicinal Chemistry*.

[110] Misra C., Sachan N., McNally C. R. (2012). Congenital heart disease-causing Gata4 mutation displays functional deficits in vivo. *PLoS Genetics*.

[111] Singh M. K., Li Y., Li S. (2010). Gata4 and Gata5 cooperatively regulate cardiac myocyte proliferation in mice. *The Journal of Biological Chemistry*.

[112] Maier H. J., Schips T. G., Wietelmann A. (2012). Cardiomyocyte-specific I*κ*B kinase (IKK)/NF-*κ*B activation induces reversible inflammatory cardiomyopathy and heart failure. *Proceedings of the National Academy of Sciences of the United States of America*.

